# Optimal Thickness Shaped Cantilever Type Vibration Energy Harvester for the Second Eigenfrequency

**DOI:** 10.3390/mi17070848

**Published:** 2026-07-17

**Authors:** Paulius Skėrys, Rimvydas Gaidys

**Affiliations:** Department of Mechanical Engineering, Kaunas University of Technology, Studentų g. 56-344, LT-51424 Kaunas, Lithuania; ktu@ktu.lt

**Keywords:** shape optimization, vibration energy harvesting, finite element method, piezoelectric energy harvesting, voltage measurement

## Abstract

Piezoelectric cantilever beams are among the most popular vibration energy harvesting devices. Maximization of the spatial distribution of axial strain along this beam (objective function) increases harvesting efficiency. In vibro-impact systems, mechanical contact can excite higher-order vibration modes, making the second eigenfrequency particularly relevant for energy harvesting under such nonlinear operating conditions. Therefore, the harvester geometry should be designed to maximize the harvested energy associated with this mode. In many practical applications, cantilever-based harvesters are subjected to complex and broadband excitation conditions, where multiple vibration modes, including the second eigenfrequency, contribute to the overall response. Therefore, optimization at the second eigenfrequency is essential for improving energy harvesting performance under realistic operating conditions. In this study, to maximize axial strain, a thickness shape optimal design is proposed, and a finite element-based optimization scheme is constructed to maximize harvesting efficiency. Optimization is performed subject to a fixed second eigenfrequency of the cantilever beam, using the eigenmode equation as the state equation in the optimization procedure. The optimized shape for maximal strain integral at the second bending resonance is determined. Experimental results validate the findings of the optimization, showing an increase in strain for the optimized-shaped beam compared to a uniform-thickness beam with the same eigenfrequency. It should be noted that experimental validation is subject to certain limitations, including manufacturing precision and environmental influences. The manufacturing of specimens can only be achieved within a limited precision, resulting in deviations from the ideal optimized geometry. Additionally, the experimental environment may influence the measured response, and simplified boundary conditions can introduce discrepancies between numerical and experimental results.

## 1. Introduction

Over the last decade, energy harvesting has become an increasingly important strategy for reducing dependence on batteries in distributed low-power electronic systems, especially where maintenance or battery replacement is impractical [1]. Comprehensive reviews highlight its relevance for wireless sensors, structural health monitoring, biomedical devices, and IoT platforms operating under limited energy budgets [2,3]. Among the available transduction mechanisms, piezoelectric energy harvesting has gained particular attention due to its solid-state operation, relatively high voltage output, and suitability for compact integration [4]. Recent materials-focused analyses further emphasize advances in functional ceramics, polymers, and multifunctional composites that continue to expand practical implementation options [5]. Broader surveys of vibration-based systems confirm that piezoelectric approaches remain one of the most widely investigated solutions in microscale and mesoscale applications [6].

Within this context, cantilever-type configurations are frequently employed because of their structural simplicity and effective strain-to-charge conversion under bending excitation [7]. Critical assessments of geometric configurations demonstrate that beam shape and dimensional tuning significantly influence energy transduction efficiency [8], while additional reviews show that electrode layout, mechanical boundary conditions, and interface circuits strongly affect overall device performance [9]. Experimental and numerical investigations into beam geometry variations further confirm that structural parameters play a decisive role in governing electromechanical coupling and output power [10]. Nevertheless, even with such refinements, harvested power levels often remain limited when real excitation amplitudes, material constraints, and damping effects are considered [11].

To address these limitations, recent research increasingly explores nonlinear and multi-stable configurations aimed at improving response under low-frequency or variable excitations [12]. Quad-stable and bistable cantilever systems have been shown to enhance dynamic response and increase usable output under broader operating conditions [13,14]. Machine-learning-assisted parameter optimization has also been introduced to refine structural performance more efficiently [15], while experimental studies continue to evaluate vibration behavior under diverse loading scenarios [16]. Alternative beam geometries, including serpentine and frequency up-conversion mechanisms, have demonstrated improved low-frequency energy capture [17,18]. Parallel advances in piezoelectric material development and structural integration further support incremental improvements in conversion efficiency [19]. Additionally, impact-driven and monostable nonlinear systems have been reported to increase harvested energy from weak ambient vibrations [20]. Collectively, these efforts illustrate how performance enhancement remains a central focus of contemporary piezoelectric energy harvesting research.

Recent research increasingly emphasizes robustness and manufacturability in topology optimization of piezoelectric energy harvesters. Rostami and Lim [21] addressed two fundamental limitations of conventional density-based methods, namely sensitivity to material and geometric uncertainties and the generation of irregular, non-manufacturable boundaries. Their robust formulation integrates uncertainty directly into the optimization problem and incorporates boundary-smoothing strategies to eliminate gray regions and jagged interfaces typical of SIMP-based approaches. The resulting designs exhibit improved strain concentration and voltage response under harmonic excitation while requiring lower computational effort than high-resolution classical schemes. By simultaneously enhancing electromechanical performance and fabrication feasibility, this work demonstrates the importance of combining robustness with geometric regularization. A similar focus on practical implementation is evident in the level-set-based framework proposed by Miyajima and Yamada [22], who explicitly incorporated manufacturability constraints into the optimization of unimorph cantilever harvesters. Their approach ensures clear material interfaces and enforces minimum feature sizes suitable for fabrication, avoiding disconnected or ambiguous structural regions. Numerical studies reported improvements in the electromechanical coupling coefficient of up to approximately 25% compared to baseline designs, alongside enhanced voltage response. These findings highlight the effectiveness of level-set methods when both performance enhancement and fabrication feasibility must be simultaneously satisfied. Further advancing fabrication-aware design strategies, Zhang, Lai, and Zhang [23] introduced an explicit topology optimization method using Moving Morphable Components (MMC) to directly parameterize geometry and polarization profiles. By incorporating graph-theoretic connectivity constraints, their framework guarantees contiguous polarization domains, which are critical for practical poling and manufacturing processes. Unlike implicit density-based approaches, this explicit formulation provides greater control over structural layout and material distribution. Numerical examples demonstrated improved energy harvesting efficiency compared to designs without enforced connectivity, confirming that explicit geometry control and polarization continuity can significantly enhance both electromechanical performance and manufacturability of optimized harvesters.

Moving from ensuring manufacturability alone to simultaneously maximizing electromechanical performance through fully coupled mechanical–electrical design formulations, recent studies have increasingly shifted toward multi-physics and multi-parameter concurrent optimization frameworks. Zhang et al. [24] exemplify this approach by proposing a concurrent topology optimization framework in which structural layout, material distribution, and polarization orientation are treated as coupled design variables. By directly integrating the mechanical and electrical governing equations and allowing polarization direction to evolve during optimization, their method enables effective redistribution of piezoelectric and elastic phases. The optimized designs demonstrated significant quantitative improvements, with output voltage and harvested power increasing by more than 30–50% compared to conventional uniform thickness configurations. These gains were primarily attributed to improved strain energy localization within active regions and enhanced phase interaction achieved through simultaneous multi-parameter optimization. Extending concurrent optimization into metamaterial systems, Pereira and Ruiz [25] developed a multi-objective framework that balances vibration suppression and energy harvesting performance. Their approach incorporates frequency-dependent dynamic analysis together with electromechanical coupling effects, allowing topology redistribution within periodic unit cells under predefined excitation bandwidths. The optimized metamaterial configurations achieved harvested power improvements of approximately 25–45% relative to non-optimized baseline structures, depending on excitation conditions and objective weighting. Importantly, the study highlights the intrinsic trade-off between vibration isolation and electrical output, demonstrating that carefully formulated multi-objective strategies can produce favorable compromises that enhance both dynamic performance and energy conversion efficiency. From a methodological perspective, Homayouni-Amlashi et al. [26] contributed a comprehensive 3D multi-material topology optimization framework that enables simultaneous optimization of material layout and polarization direction within a fully coupled electromechanical finite element model. By extending previous 2D formulations to three dimensions and incorporating the PEMAP-P interpolation scheme, their implementation allows more realistic representation of spatial interactions between active and passive phases. Case studies confirmed improved strain distribution and enhanced electromechanical response in optimized configurations, while the open MATLAB codes facilitate broader adoption of high-fidelity concurrent optimization approaches. Complementarily, Hu et al. [27] addressed broadband performance by formulating a multi-objective topology optimization problem that maximizes power output at two adjacent eigenfreencies. Their optimized designs achieved approximately 60% higher average power across the target bandwidth compared to standard cantilever geometries, demonstrating that concurrent multi-frequency optimization can effectively overcome the narrowband limitation typical of conventional piezoelectric harvesters.

While concurrent multi-physics optimization strategies significantly expand the design space, meaningful performance improvements can also be achieved through purely geometric or parametric shape optimization of cantilever-type harvesters. In this line of research, the structural topology remains fixed, and performance enhancement is pursued by systematically tailoring beam geometry to improve strain distribution and electromechanical coupling. Hasani and Shahverdi [28] investigated the shape optimization of a non-uniform parametric piezoelectric cantilever beam under harmonic excitation. Instead of adopting the conventional rectangular configuration, they introduced a variable width distribution along the beam length and optimized its geometric profile to enhance strain concentration within the piezoelectric layer. The optimization targeted maximum electrical power output while satisfying structural and geometric constraints. Their results showed that non-uniform beam geometries significantly outperform standard prismatic designs, with reported power output increases of approximately 20–40% compared to a reference rectangular cantilever. The improvement was primarily attributed to more effective strain localization near the clamped end, demonstrating that carefully controlled geometric modification alone can substantially increase energy harvesting efficiency. Similarly, Salman, Lustig, and Elata [29] analytically derived the optimal planform shape of a unimorph cantilever harvester equipped with a device-layer edge block. Using beam theory and strain uniformity criteria, they obtained closed-form solutions leading to curved beam contours defined through Bessel-type functions. The optimized planforms redistribute bending stresses more uniformly along the beam length, thereby enhancing electromechanical conversion efficiency. Comparative analyses revealed performance gains in the range of approximately 15–30% in terms of voltage response and harvested power relative to conventional rectangular geometries. This study highlights that analytical planform shaping, even without full topology redistribution or material modification, can significantly improve strain uniformity and electrical output in piezoelectric cantilever harvesters.

Beyond deterministic geometric optimization, recent studies have incorporated data-driven approaches to efficiently explore complex design spaces. Kim and Lee [30] developed a deep neural network (DNN)-assisted shape optimization framework for gradient-index phononic crystals aimed at enhancing piezoelectric energy harvesting. By using a surrogate DNN model to predict wave focusing behavior, the optimization process was significantly accelerated while enabling improved structural designs. The optimized configurations achieved 1.5–2.0 times higher focused elastic wave intensity compared to baseline layouts, resulting in enhanced vibrational energy transfer to the piezoelectric element and improved electromechanical conversion efficiency.

While many studies focus on optimizing the fundamental resonance, recent research increasingly investigates the second eigenfrequency to enable multiple dynamic modes to contribute effectively to energy harvesting under realistic multi-frequency excitation. Gibus et al. [31] addressed this challenge through an analytical design methodology for two-degree-of-freedom (2-DOF) piezoelectric cantilevers capable of operating efficiently at both the first and second resonances. Their closed-form model enables systematic tuning of proof mass and electrode geometry to control the spacing between the two eigenfrequencies. A validated prototype exhibited closely spaced resonances at 35.3 Hz and 53.2 Hz, achieving electromechanical coupling coefficients of approximately 10.4% for the first mode and 8.7% for the second. Under 0.5 m/s^2^ excitation, the device produced 262 µW at the first resonance and 149 µW at the second, demonstrating that the second eigenmode can meaningfully contribute to total harvested energy and significantly broaden operational bandwidth. Complementarily, Yang and Chen [32] analyzed how geometric parameters affect higher modal behavior, including the second mode, within a magnetic-coupled negative-stiffness harvester. They showed that increasing beam thickness by 300% raised the second characteristic frequency by approximately 40.4%, while width changes showed an increase of approximately 429 Hz increase in the second mode when expanded from 6 mm to 10 mm. Their findings highlight that the second mode responds differently to geometric variation than the first mode and must be considered explicitly when designing broadband or multi-mode systems. Similarly, Vyas et al. [33] demonstrated that a micromachined M-shaped coupled-cantilever structure can intentionally exploit two closely spaced bending modes to generate dual resonance peaks. In this configuration, the second eigenfrequency is no longer an unused higher-order response but an active contributor to energy harvesting, increasing bandwidth and reducing sensitivity to excitation variability. Finite element and experimental results confirmed improved stress distribution and enhanced operational range compared to conventional single-cantilever harvesters. Collectively, these studies underscore that deliberate investigation and tuning of the second eigenfrequency is essential for overcoming narrowband limitations and achieving more stable, broadband energy harvesting performance.

Researchers’ attention has also shifted toward harvesting concepts that combine multiple excitation sources and transduction mechanisms within a single device architecture. In this context, Dong et al. [34] proposed a dual-mode magneto-mechano-electric harvester that exploits distinct bending modes to simultaneously capture vibration and magnetic field energy, demonstrating that modal coupling and mode-specific design can significantly enhance overall system performance. Similarly, Dong et al. [35] introduced an aerodynamics-driven nanogenerator leveraging galloping–flutter synergy, where dynamic interaction between different flow-induced vibration modes was optimized to achieve high efficiency across a broad range of wind speeds. Complementing these developments, Gao et al. [36] designed a hybrid triboelectric–electromagnetic–piezoelectric system, in which structural integration and multi-physics coupling enable simultaneous harvesting of wind and vibration energy while maintaining vibration attenuation capabilities. A related hybrid approach was presented by Yong et al. [37], who developed a self-adaptive nanogenerator with dual-channel power management, demonstrating that coordinated structural and electrical optimization can ensure stable energy output under varying environmental conditions.

In addition to hybrid system integration, Vibro-shock and impact-based piezoelectric energy harvesters have also been investigated as effective solutions for converting low-frequency excitation into higher-frequency structural response. Žižys et al. [38] analyzed a tandem system consisting of a low-frequency resonator and a high-frequency piezoelectric harvester, showing that impact coupling, natural-frequency ratio, and contact-point location strongly influence transient power output and higher-mode activation. In a related study, Žižys et al. [39] examined a vibro-shock cantilever-type harvester operating in higher transverse vibration modes and demonstrated that accurate segmentation at the strain node improves effective strain contribution and voltage output. More recently, Peng et al. [40] proposed a frequency up-conversion piezoelectric harvester with a low-frequency oscillator and stop limiter, where collision-induced nonlinear force was used to broaden the operating bandwidth and enhance high-frequency electromechanical response. Wang et al. [41] further extended vibro-impact harvesting by introducing an acoustic-black-hole beam configuration, where contact dynamics, impact position, and thickness-dependent energy localization were shown to affect the output response. Liu et al. [42] developed a dual-impact strategy for small acceleration amplitude vibrations, demonstrating improved peak voltage, dual-band frequency response, and experimentally validated nonlinear coupled dynamics. These studies demonstrate that vibro-shock and impact-based excitation can activate higher vibration modes, redistribute strain along the cantilever, and improve energy conversion under non-smooth dynamic conditions; however, they also indicate that the role of thickness profile optimization in controlling modal strain distribution remains insufficiently addressed.

Recent studies have also focused on multimode and broadband optimization strategies aimed at overcoming the narrow operating bandwidth of conventional piezoelectric harvesters. In this regard, Gibus et al. [31] proposed an analytical design methodology for two-degree-of-freedom piezoelectric cantilevers capable of harvesting energy at multiple resonances. By introducing an additional degree of freedom, the authors achieved two closely spaced vibration modes and demonstrated that higher-order modes, including the second eigenfrequency, can contribute significantly to the harvested energy and broaden the operational bandwidth. A complementary optimization approach was presented by Hu et al. [27], who developed a multi-objective topology optimization framework for broadband piezoelectric energy harvesters. Their methodology simultaneously considered the electromechanical response at multiple eigenfrequencies, enabling improved power generation across a wider excitation frequency range compared with conventional single-mode designs. These studies demonstrate that multimode operation and broadband optimization represent promising directions for improving energy harvesting performance under realistic excitation conditions.

Although significant progress has been made in the design and optimization of piezoelectric energy harvesters, the potential of tailoring non-linear thickness distributions for targeted eigenfrequencies remains relatively underexplored. Most existing studies focus on width variation or topology optimization, while thickness is typically kept uniform or modified without explicitly addressing its influence on modal strain energy distribution. Since piezoelectric coupling is most effective when regions of high mechanical strain coincide with polarized material, shaping the thickness profile offers a promising pathway for performance enhancement. In this study, the electromechanical response of a cantilever-type piezoelectric energy harvester is improved by optimizing its thickness distribution while maintaining the second natural frequency, using a combined numerical and experimental framework that includes gradient-based optimization, finite element modeling, and experimental validation.

## 2. Theory

This section provides the analysis encompasses the Euler–Bernoulli beam model, focusing on its mathematical and numerical implementation via a finite element method (FEM). Modeling of the substructure element is carried out using the beam finite element.

**Figure 1:**
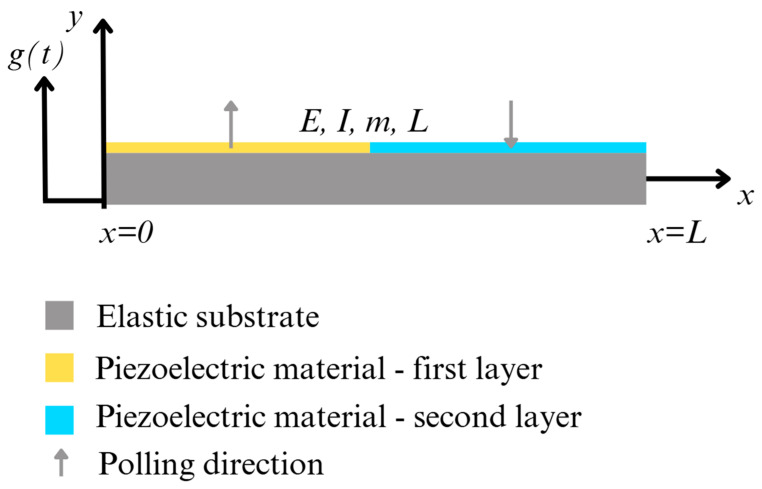
Configuration of the segmented Piezoelectric Beam.

The section further addresses the principles of undamped free vibration, presenting the mathematical background and equations of state necessary to optimize the strain distribution in cantilever beam. An integral part of the design of modern vibration energy harvesters is their optimal design, which makes it possible to create structures that meet the set requirements. A typical piezoelectric beam structure (Figure 1) is composed of one or more piezoelectric layers bonded to a substrate with an electrical interface. In order to design the optimal shape of the substructure element, a gradient projection method in state space was used. The problems of optimal design of the cantilever type vibration energy harvester for fixed second eigenfrequency of transversal oscillations of substructure element are considered.

This section provides an investigation of the core principles underlying the theory of linear piezoelectricity as it pertains to thin beam structures. This analysis provides a comprehensive examination of the behaviour of piezoelectric materials, illustrating the coupling between mechanical and electrical properties. This discussion also covers modelling of a cantilever beam under base excitation, emphasizing key concepts such as axial strain.

### 2.1. Model of Substructure

**Figure 2:**
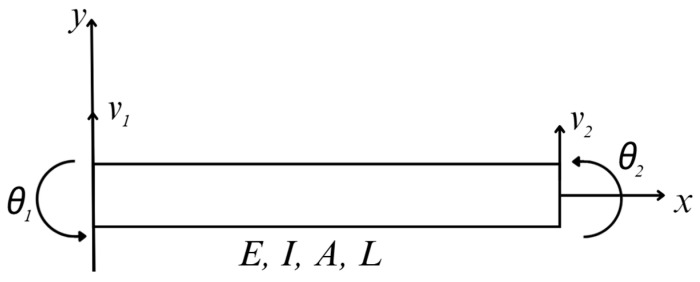
A typical beam element with nodal displacements in a local reference frame.

The simplified Bernoulli–Euler beam element is characterized by two degrees of freedom (DOFs), as illustrated in Figure 2. In this figure node displacements are shown in a local coordinate system.

The displacement field for a finite element can be represented as:

(1) f=Nr,

where f is a vector function, N is a vector of known functions, and r is a vector of generalized nodal displacements for an element. Dimensions of the vector N and the vector r depend on the degree of freedom of the element. The vector N is referred to as the shape function.

For the beam element of Figure 2, the displacement function is f=v(x,y). The shape function N(x,y) is obtained by solving the beam differential equation. Neglecting shear deformation effects, the shape function is given as [43]:

(2) $$N=\left[\begin{array}{llll} 6\left(\xi -\xi^{2}\right)\eta & -\left(1-4\xi +3\xi^{2}\right)\eta L & -6\left(\xi -\xi^{2}\right) & \left(2\xi -3\xi^{2}\right)\eta L \end{array}\right],$$

where ξ=x/L and η=y/L.

The displacement field of (1) is used to obtain the strains:

(3) ε=Br,

where ε is strains and B is a vector that is obtained from differentiation of terms in the matrix N. Thus, (3) expresses the strain field for the element in terms of the generalized nodal displacements r.

For the beam element of Figure 2, there is only axial strain $\left(\varepsilon_{x}=\partial u/\partial x\right)$ due to the elementary beam theory, and the matrix B is identified as:

(4) $$B=\frac{1}{L}\left[\begin{array}{lll} 6(1-2\xi )\eta & 2(2-3\xi )\eta L & 6(2\xi -1)\eta \end{array}2(1-3\xi )\eta L\right],$$

or

(5) $$\left[B\right]=-y\left[-\frac{6}{L^{2}}+\frac{12x}{L^{3}}\;\;\;\;-\frac{4}{L}+\frac{6x}{L^{2}}\;\;\;\;\;\;\;\frac{6}{L^{2}}-\frac{12x}{L^{3}}\;\;\;\;\;\;-\frac{2}{L}+\frac{6x}{L^{2}}\right],$$

and

(6) $$\eta =\left\{\begin{array}{c} V_{1}\\ \theta_{1}\\ \begin{array}{c} V_{2}\\ \theta_{2} \end{array} \end{array}\right\},$$

(7) $$\varepsilon =-y\left[-\frac{6}{L^{2}}+\frac{12x}{L^{3}}\;\;\;\frac{6}{L^{2}}-\frac{12x}{L^{3}}\;-\frac{4}{L}+\frac{6x}{L^{2}}\;\;-\frac{2}{L}+\frac{6x}{L^{2}}\right]\left\{\begin{array}{l} v_{1}\\ \theta_{1}\\ v_{2}\\ \theta_{2} \end{array}\right\}.$$

The equilibrium equations for an element can now be obtained from the principle of virtual work, or stationary potential energy. Writing the virtual work of all the forces and equating it to zero, one has

(8) $$\int_{V}\;\;\delta \varepsilon \sigma dV-\delta r^{T}p=0$$

where p is a vector of generalized nodal forces that correspond to the nodal displacements r. The generalized Hooke’s law is used to express stress-strain,

(9) σ=Dε,

where σ is an axial stress and D is an elastic constant. Substituting (3) and (9) into (8), the principle of virtual work is expressed as:

(10) $$\delta r^{T}\left[\left(\int_{V}\;\;B^{T}DBdV\right)r-p\right]=0,$$

Since all components of δr are arbitrary, (10) is satisfied only when the term in square brackets is zero, or,

(11) p=kr,

where the matrix k is given as:

(12) $$k=\int_{V}\;\;\left(B^{T}BD\right)dV\;,$$

For the beam elements of Figure 2, the matrix D is simply the scalar D=E. Substituting appropriate matrices B and D into (12) and carrying out the indicated integration, one obtains element stiffness matrix for the beam element as:

(13) $$k=\frac{EI}{L^{3}}\left[\begin{array}{rrrr} 12 & 6L & -12 & 6L\\ & 4L^{2} & -6L & 2L^{2}\\ & & 12 & -6L\\ Symmetric\; & & & 4L^{2} \end{array}\right].$$

The mass matrix for an element is computed by its kinetic energy as a quadratic form in generalized velocities. The kinetic energy of an element is,

(14) $$KE=\frac{1}{2}\int_{V}\;\;{\dot{f}}^{T}\dot{f}\rho dV,$$

where ρ is the mass density. Substituting for f from (1), in terms of the shape function *N*, obtains,

(15) $$KE=\frac{1}{2}{\dot{r}}^{T}m\dot{r},$$

where m is the element mass matrix, which is given as,

(16) $$m=\int_{V}\;\;\rho N^{T}NdV,$$

From (16) and the shape function from (2), the mass matrix for beam element is

(17) $$m=\frac{\rho AL}{420}\left[\begin{array}{rrrr} 156 & 22L & 54 & -13L\\ & 4L^{2} & 13L & -3L^{2}\\ & 156 & & -22L\\ Symmetric & & & 4L^{2} \end{array}\right].$$

The matrix (17) represents translational inertia of the beam element.

### 2.2. Equations of Motion

Equations of motion for a structural system are derived using Hamilton’s principle [44]. For the linearly elastic structure with small displacements, the equation of motion is given as:

(18) $$M\ddot{z}+Kz=S\left(t\right),$$

where S(t) is a forcing function. For free harmonic motion of the structure z=ysinωt, where y is an eigenvector and ω is a natural frequency. Substituting this into (15), with S(t)=0 and defining $\zeta =\omega^{2}$, one obtains

(19) Ky=ζMy.

Equation (19) is the generalized eigenvalue problem that must be solved for natural frequencies and the corresponding mode shapes (the eigenvectors).

### 2.3. Finite Dimensional Optimal Design for Maximization the Axial Strain of the Cantilever

In most problems of engineering design, the system being designed is required to behave according to some law of physics. This behavior is described analytically by a set of variables called state variables. Further, there is a second set of variables that describe the system, rather than its behavior. These variables are called design variables since they are to be chosen by the designer and serve as to assure the specifications for fabrication. The equations that determine the state of mechanical and structural systems generally depend on the design variables, so the two sets of variables are related.

The following design problem is considered: determine the size of beam thickness to be used in a structure so that a natural frequency of the structure is within the given limits, the thickness of certain points on the structure is within the given limits, and the structure layer integral of the normal strain is as high in value as possible.

The beam thickness is one of the design variables in this problem, since it describes the structure being designed, and it must be chosen by the designer. Eigenfrequency is a state variables that is determined by equilibrium relation of the generalized eigenvalue problem. Again, the designer has no direct control over natural frequency. They may affect this quantity only by varying the thickness of beam in the structure.

The gradient method, which relies solely on first derivative (gradient) information, is used to iteratively improve the estimated solution. Geometrically, this method first determines the direction of the most rapid increase in the cost function $\psi_{0}(b)$; this is $\nabla \psi_{0}^{T}(b)$, where b is vector of design variables. This direction is then projected onto the tangent hyperplane to the boundary of the constraint set at the design b. A small move in the resulting direction will then increase $\psi_{0}(b)$ and will not cause excessive violation of constraints. This process is repeated as long as $\psi_{0}(b)$ can be increased.

The method is based on a state space formulation of mechanical system problems, in terms of matrix equations. A joint equation is used to define a set of variables that provide explicit design sensitivity information.

The idea of sensitivity analysis is an approximation of the problem that can be analyzed to determine the effect of the change δb in $b^{0}$. Linear approximations to the changes in $\psi_{0}(b)$ and $\psi_{j}(\zeta ,b)$ due to the small changes in the variables, are:

(20) $$\delta \psi_{0}\left[b^{0}\right]=\frac{\partial \psi_{0}}{\partial b}\left[b^{0}\right]\delta b,$$

and,

(21) $$\delta \psi_{k}\left[\zeta^{0},b^{0}\right]=\frac{\partial \psi_{k}}{\partial \zeta }\left[\zeta^{0},b^{0}\right]\delta \zeta +\frac{\partial \psi_{k}}{\partial b}\left[\zeta^{0},b^{0}\right]\delta b.$$

The symbol δ in front of a function denotes the total differential of the function.

First-order changes in y and ζ may be analyzed. Premultiplying (16) by $y^{T}$, one has the identity $y^{T}K(b)y=\zeta y^{T}M(b)y$. Approximating both sides to first order in the variables y,ζ, and b, one has:

(22) $$\delta \zeta =\left[\frac{\partial }{\partial b}\left\{y^{T}K(b)y\right\}-\zeta \frac{\partial }{\partial b}\left\{y^{T}M(b)y\right\}\right]\delta b\equiv l^{\xi^{T}}\delta b.$$

This is an explicit relationship that determines the change in the eigenvalue δζ in terms of the change δb in design [45]. Equation (22) can be substituted into (21) to express δζ in terms of δb:

(23) $$\delta \Psi_{k}=\frac{\partial \Psi_{k}}{\partial \zeta }l^{\zeta T}\delta b+\frac{\partial \Psi_{k}}{\partial b}\delta b.$$

Column $l^{k}$ of matrix l is as follows:

(24) $$l^{k}=\frac{\partial \Psi_{k}^{T}}{\partial b}+\frac{\partial \Psi_{k}^{T}}{\partial \zeta }l^{\zeta }.$$

Column $l^{k}$ represents the sensitivity coefficients of the k-th constraint $\Psi_{k}$ with respect to the design variables and consists of the derivatives of this constraint. Element $l_{j}^{k}$ is the derivative of $\Psi_{k}$ with respect to the j-th design variable. Thus, if $l_{j}^{k}>0$, an increase in $b_{j}$ will lead to an increase in $\Psi_{k}$; if $l_{j}^{k}<0$, an increase in $b_{j}$ will result in a decrease in $\Psi_{k}$.

The optimization problem is formulated as follows:

Find $DV_{j}$ to:

(25) $$max\int_{0}^{L}\left|\;\varepsilon dx\right|=\sum_{j=1}^{N}\left|{\;B}^{j}r^{j}\right|,$$

state equation,

(26) K(DV)y=ζM(DV)y,

(27) $${DV}_{min\;}\leq DV_{j}\leq {DV}_{max},j=1,\;N,$$

(28) $${\zeta_{2}}_{min}\leq \zeta_{2}\leq {\zeta_{2}}_{max},$$

where:

N—number of design variables,

E—normal strain at the layer of the beam,

$DV_{j}\;-j$th design variable,

$\zeta_{2}$—second natural frequency,

y—eigen form matrix,

B—strain-displacement matrix,

r—vector of nodal displacements.

During the optimization procedure, the status of the imposed constraints is evaluated at every iteration. If a constraint becomes active, or approaches its admissible limit, the optimization algorithm must account for this condition in order to prevent violation of the feasible design space. For this reason, sensitivity analysis is also carried out at each iteration. The derivatives with respect to the design variables are obtained according to Equation (24). For the eigenfrequency constraints defined in Equation (28), the derivatives with respect to the second eigenvalue variable have straightforward analytical expressions. For the lower and upper eigenfrequency bounds, respectively, these derivatives are written as $\frac{\partial \Psi_{k}}{\partial \zeta_{2}}=-1/{\zeta_{2}}_{\min}$ and $\frac{\partial \Psi_{k}}{\partial \zeta_{2}}=1/{\zeta_{2}}_{\max}$, respectively. Therefore, the vector $l^{\zeta k}$ introduced in Equation (22) defines the contribution of the eigenvalue constraint sensitivity. Using Equation (24), the derivative of the constraint function with respect to the design variables can then be expressed as,

(29) $$\delta \zeta_{2}=l^{\zeta_{2}T}\delta b.$$

The gradient vector of the eigenvalue in this case takes the form

(30) $$l^{\zeta_{2}}={\left[\frac{\partial \left(K\varphi_{2}\right)}{\partial b}-\zeta_{2}\frac{\partial \left(M\varphi_{2}\right)}{\partial b}\right]}^{T}\varphi_{2}.$$

**Figure 3:**
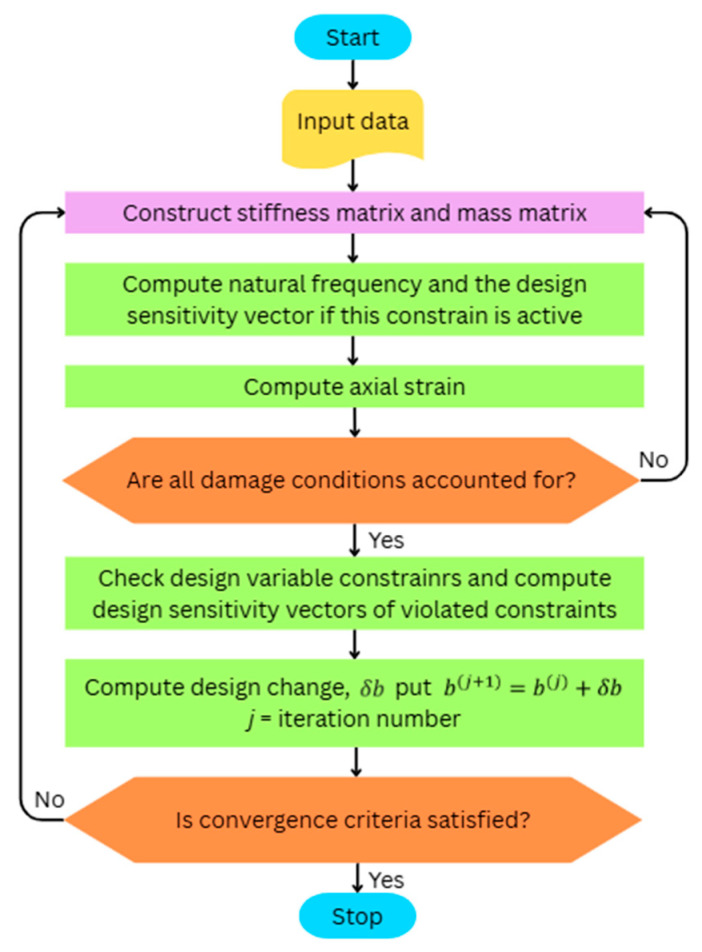
The flowchart of the optimization process.

A simplified flow diagram is given in Figure 3. 

Functionally, this program consists of three major elements. The first is for analysis of the structure, checking of constraints, and construction of sensitivity vectors. The second element is for computation of the projected direction of steepest descent and constraint corrections. The third element is a check of convergence criteria.

In the problem treated, the objective function is taken as a structure layer integral value of the normal strain, which is a function of design and state variables.

The formulated optimization problem was solved (MATLAB R2024b) using the gradient projection method in state space, and optimal shape design of the cantilever for fixed second natural frequency with the maximized normal strain was found.

### 2.4. Model of Unimorph Cantilever-Type Piezoelectric Beam

The widely accepted piezoelectric energy harvester, a cantilever-type harvester, is explained in this section. It utilizes the piezoelectric effect to generate electric potential in response to the applied mechanical strain.

**Figure 4:**
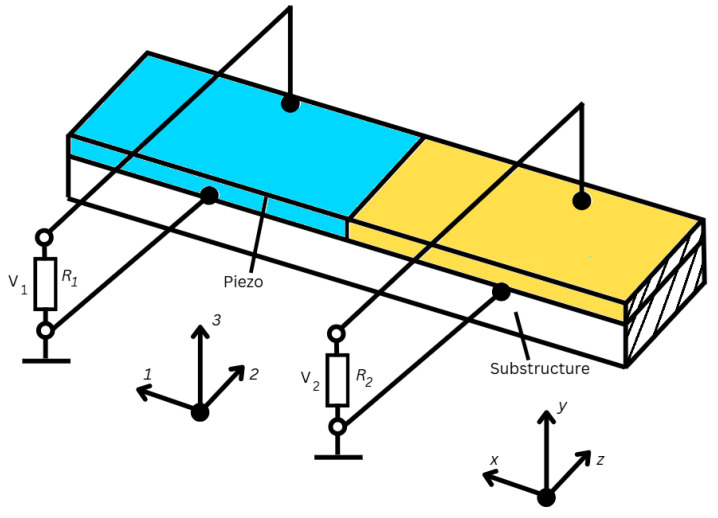
Unimorph cantilever consisting of passive substrate and a piezoelectirc layer: blue—first, yellow—second. The hatched area marks the mechanical clamping which constrains all translational degrees of freedom. The piezo material is covered by electrodes on its top area.

A piezoelectric unimorph is a cantilever-type harvester composed of one piezoelectric layer shown in Figure 4. 

The piezoelectric constitutive behavior is adopted considering a thin beam, based on the Euler-Bernoulli assumptions. The analytical approach, used for the development of the dynamic model, adopts the variational indicator proposed in the works of [46,47]:

(31) $$(VI)=\int_{t_{1}}^{t_{2}}\;[\delta K-\delta P+f\delta x]dt=0,$$

where K is the kinetic energy of the beam, P is the elastic energy of the beam, and fδx is the external work applied to the system, having f as the external force. Here, P is defined as the electric enthalpy H(S,E) due to the electromechanical coupling. The definitions of K,P, and fδx are given as:

(32) $$K=\frac{1}{2}\int_{V_{s}}\;\;\rho_{s}{\dot{u}}^{T}\dot{u}dV_{s}+\frac{1}{2}\int_{V_{p}}\;\;\rho_{p}{\dot{u}}^{T}\dot{u}dV_{p},$$

(33) $$P=\frac{1}{2}\int_{V_{s}}\;\;S^{T}\sigma dV_{s}+\frac{1}{2}\int_{V_{p}}\;\;S^{T}\sigma dV_{p}-\frac{1}{2}\int_{V_{p}}\;\;E^{T}DdV_{p},$$

(34) $$f\delta x=\sum_{i=1}^{n_{f}}\;\;\delta u\left(x_{i}\right)\cdot f_{i}\left(x_{i}\right)-\sum_{j=1}^{n_{q}}\;\;\delta v\cdot q_{j}.$$

where S and σ are the mechanical strain and stress, E and D are the electrical potential and displacement, v and $q_{j}$ are the applied voltage and charge, $u\left(x_{i}\right)$ is the displacement along the position $x_{i}$ of the beam, ρ is the density, and the subscripts s and p prefer the substrate and piezoelectric materials, respectively. For a single-degree-of-freedom cantilever equivalent beam model, the constitutive piezoelectric equation is given by (35) in strain-charge form:

(35) $$S_{1}=s_{11}^{E}\sigma_{1}+d_{31}E_{3},\;D_{3}=d_{31}\sigma_{1}+\varepsilon_{33}^{\sigma }E_{3},$$

where $s_{11}^{E}$ is the compliance under constant electric field, $d_{31}$ is the piezoelectric constants property, and $\varepsilon_{33}^{\sigma }$ is the dielectric constant at constant stress. Therefore, the only non-zero stress is $\sigma_{1}$, implying that bending yields strains in the 1st direction only, which polarizes the surface perpendicular to the direction of the applied stress, i.e., the 3rd direction. Having defined the constitutive piezoelectric equations, one updates (32)–(34) by incorporating the coupled relationship to them. It is considered that the beam undergoes strain in x-direction only, represented by $S_{1}$, and polarization in z-direction, represented by $E_{3}$. Therefore, adopting the Euler-Bernoulli beam theory, the strain along the beam is defined as:

(36) $$S_{1}(x,t)=-t_{pc}\frac{\partial^{2}u(x,t)}{\partial x^{2}},$$

where $t_{pc}$ is the distance between the symmetry axis of the beam to the center of the piezoelectric layer. The assumption that the electric potential is uniform across the thickness of the piezoelectric layer ($t_{p}$) allows simplifying the electric field as follows:

(37) $$E_{3}=-\frac{v(t)}{t_{p}}.$$

## 3. Results

To improve the electromechanical efficiency of the piezoelectric energy harvester operating at the second bending eigenfrequency, the geometry of the cantilever beam was optimized by modifying the thickness distribution of the elastic substrate while constraining the structure to maintain the target dynamic characteristics. The primary design objective was to maximize the strain generated in the upper surface of the beam, where the piezoelectric layer is attached, thereby increasing electrical polarization and the resulting energy output. At the same time, the optimization ensured that the cantilever retained the required resonance frequency so that the optimized structure remained compatible with the intended operating conditions of the harvester.

The base geometry consisted of a cantilever beam with a length of 100 mm and a uniform width of 10 mm. Verification of FEM mesh independence was performed with 10, 20, and 40 FE. Integral value of the normal strain in the upper layer of the optimal shape cantilever for corresponding FE mesh is: 6.95 × 10^−8^ m, 7.4 × 10^−8^ m, and 7.56 × 10^−8^ m. Relative error between 10 and 20 FE mesh is 0.06, and between 20 and 40 FE mesh is 0.02. Once the results of the 20 FE mesh are flattened out, the solution is considered “mesh independent”. The eigenfrequency is the state variable in the optimal design problem and is constrained by the given value. Finite element modeling (FEM) was used for structural optimization, employing 20 beam-type elements to discretize the beam along its length. The thickness of the beam was constrained to remain above a minimum allowable value, while the optimization process was carried out under the requirement that the second eigenfrequency remained close to the target value of approximately 270 Hz. These constraints ensured that the optimization process enhanced the strain generation capability of the cantilever while preserving the desired dynamic behavior.

**Figure 5:**
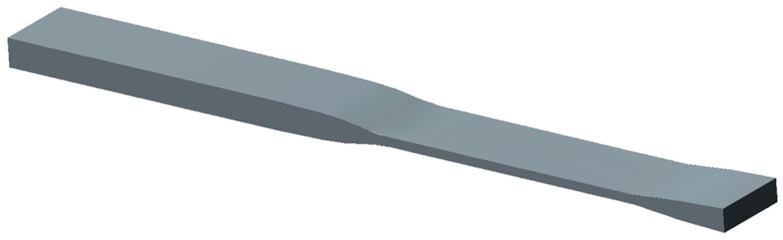
Substructure of the optimal thickness profile for the cantilever optimized for the second eigenfrequency (thickness scale exaggerated ×4 relative to beam length).

The initial design consisted of a cantilever beam with uniform thickness along its entire length. The optimized geometry obtained through the numerical procedure significantly deviates from this uniform thickness profile, producing a nonuniform thickness distribution ranging from 1.45 mm to 2.33 mm. The resulting optimal thickness profile is presented in Figure 5.

The optimized cantilever can be divided into three functionally distinct structural segments. The first segment begins at the clamped end of the cantilever and extends to approximately 0.54 L of the total beam length. In this region, the thickness reaches its maximum value of approximately 2.33 mm. Because this segment is located near the fixed support, it experiences relatively small deformation and mainly contributes to the structural stiffness and stability of the system. The second segment extends from approximately 0.54 L to 0.84 L and gradually narrows to the minimum thickness of about 1.45 mm. This section forms the primary flexible region of the cantilever and generates the largest portion of the mechanical strain experienced by the upper surface of the beam. The third segment occupies the remaining portion of the cantilever from 0.84 L to the free end. In this region, the thickness increases nonlinearly from the thinnest part of the beam to approximately 2.1 mm, effectively forming an inertial extension that increases the dynamic response of the structure near resonance.

**Figure 6:**
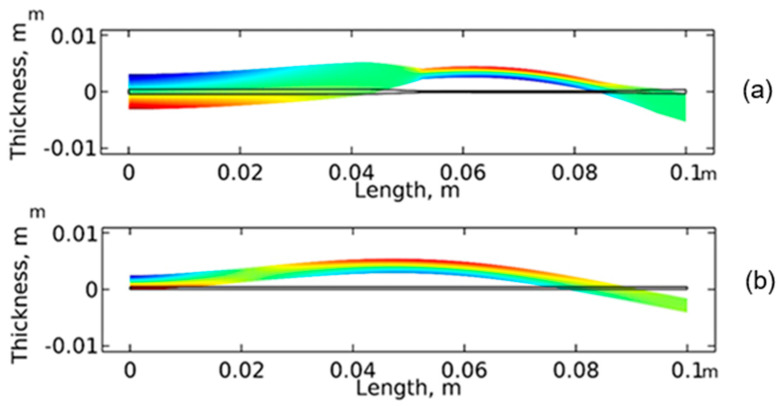
The second eigenform of the deformed cantilever and its normalized normal strain field: (**a**) optimized thickness profile; (**b**) constant thickness beam.

To evaluate how this optimized geometry influences the mechanical behavior of the cantilever, the normal strain distribution corresponding to the second vibration mode was calculated. The resulting strain field is illustrated in Figure 6, where the strain field of the optimized cantilever is compared with that of the uniform thickness beam configuration.

For the uniform thickness cantilever, the strain distribution follows the typical behavior of a beam vibrating in the second bending mode, where the deformation changes sign along the beam length and a nodal point appears between two regions of opposite strain. In contrast, the optimized cantilever exhibits a modified strain distribution resulting from the tailored stiffness and mass distribution. The thicker segment near the clamped boundary stabilizes the structure, while the thinner central segment produces a relatively uniform strain region that is particularly suitable for energy conversion.

**Figure 7:**
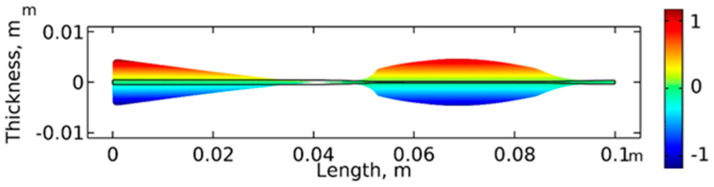
Normalized normal strain distribution along the optimized thickness cantilever under second-mode vibration.

A more detailed representation of the strain distribution along the optimized cantilever is presented in Figure 7.

**Figure 8:**
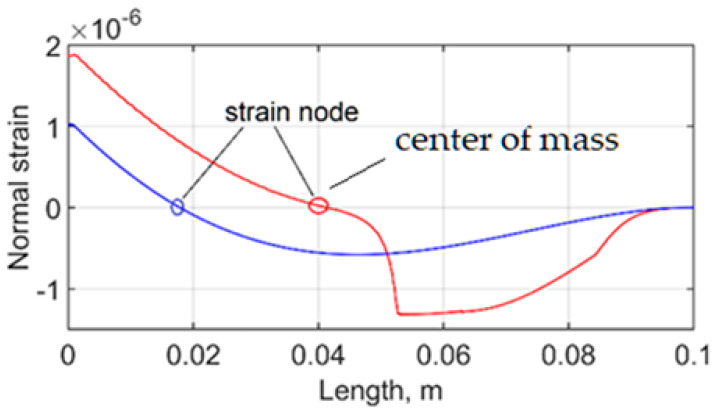
Normal strain distribution at the upper layer of the cantilever, kinematically excited by second eigenfrequency, during transient analysis at the time moment of the max strain (analogues to eigenform). Blue—uniform thickness cantilever, red—optimized design cantilever.

The transient analysis further clarifies how the optimized geometry redistributes strain along the cantilever when the structure vibrates at the second eigenfrequency. The normal strain distribution corresponding to the moment of maximum strain is presented in Figure 8. The blue curve represents the uniform thickness cantilever, whereas the red curve corresponds to the optimized design. The results show that the optimized cantilever generates substantially higher strain over most of the beam length compared with the uniform thickness configuration. This improvement is particularly noticeable in the regions close to the clamped boundary and toward the free end, where the modified stiffness and inertia distribution amplify the deformation of the beam.

The strain field reveals the presence of a nodal point located at approximately 0.41 L of the cantilever length, where the strain changes sign. This divides the cantilever into two regions of opposite deformation. Because the piezoelectric layer generates electrical charge proportional to the strain in the substrate, a continuous electrode across this node would produce charges of opposite polarity that partially cancel each other. For this reason, the piezoelectric layer must be segmented at the nodal location. As a result, the optimized cantilever effectively behaves as two coupled cantilever sections oscillating in partial counterphase, which enhances the overall strain generation and improves the energy harvesting capability at the second eigenfrequency. Furthermore, the shift of the strain node coincides with the effective center of mass of the optimized substructure, leading to a more dynamically balanced configuration. This alignment reduces undesired inertia moments and minimizes internal dynamic inconsistencies during vibration, resulting in a more efficient transfer of mechanical energy into usable strain. 

The comparison also highlights a significant shift in the location of the strain node associated with the second vibration mode. For the uniform thickness cantilever, the node appears at approximately 0.18 L along the beam length. In contrast, for the optimized design, the node moves to approximately 0.41 L. This relocation is directly related to the nonuniform thickness distribution introduced during the optimization process.

Importantly, the strain node of the optimized cantilever coincides with its center of mass, which contributes to a more balanced dynamic behavior of the structure during vibration. This alignment improves the efficiency of the strain distribution and supports the effective operation of the cantilever in the second vibration mode.

**Figure 9:**
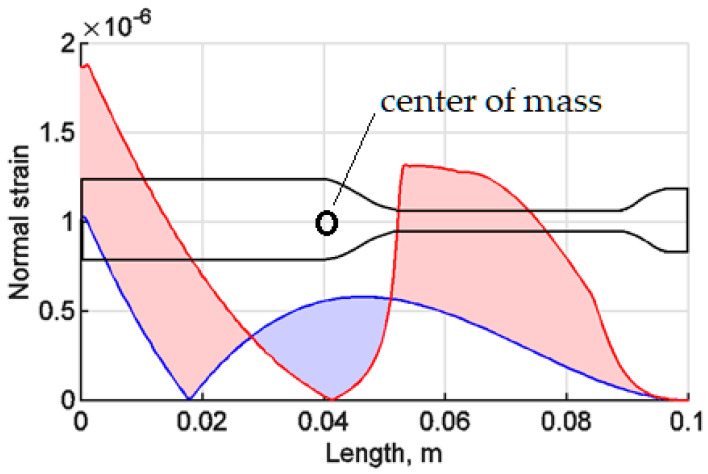
Normal strain generated in the upper layer of the cantilevers (kinematically excited at the second eigenfrequency) obtained from transient analysis at time of the maximum normal strain generated by the cantilevers. Blue—uniform thickness cantilever; red—optimized cantilever design. The shading represents where optimized design is superior (red) and where the uniform thickness cantilever is producing more strain (blue). Black—optimal shape of the cantilever.

A more detailed comparison of the strain generation along the beam is shown in Figure 9. 

The plot illustrates the integral distribution of the normal strain along the cantilever length at the moment corresponding to the maximum strain obtained from the transient analysis. The red curve represents the optimized cantilever, while the blue curve corresponds to the uniform thickness beam configuration.

The results demonstrate that the optimized cantilever produces significantly larger strain values across most of the beam. In the region extending from the clamped boundary to the nodal point at 0.41 L, the optimized design generates an integrated strain value of 3.2 × 10^−8^ m, whereas the corresponding region of the uniform thickness cantilever produces only 8.74 × 10^−9^ m. In the second segment of the beam, extending from the node to the free end, the optimized cantilever generates 4.04 × 10^−8^ m, compared with 2.7 × 10^−8^ m for the uniform thickness design.

**Figure 10:**
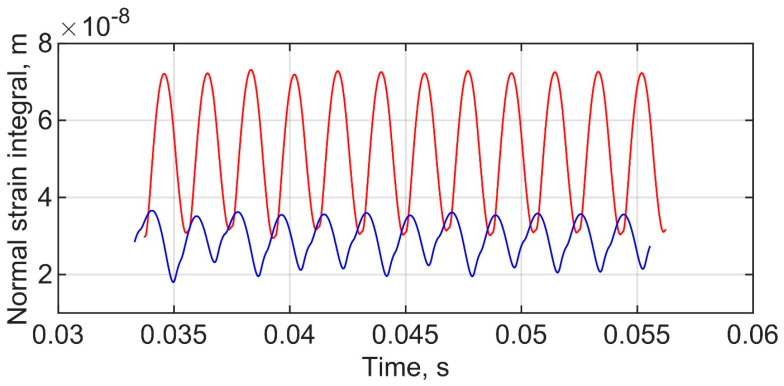
Integral of the normal strain in the upper layer of the cantilevers kinematically excited at the second eigenfrequency. Blue—uniform thickness thickness cantilever; red—optimal design cantilever.

The overall effect of this redistribution is illustrated in Figure 10, which presents the integral value of the normal strain generated in the upper layer of the cantilevers during vibration at the second eigenfrequency. The blue curve corresponds to the uniform thickness cantilever, while the red curve represents the optimized design. The optimized cantilever reaches a maximum strain integral of 7.4 × 10^−8^ m, whereas the uniform thickness beam produces 3.6 × 10^−8^ m, confirming that the optimized thickness distribution significantly increases the total strain generated in the cantilever during resonant vibration.

**Figure 11:**
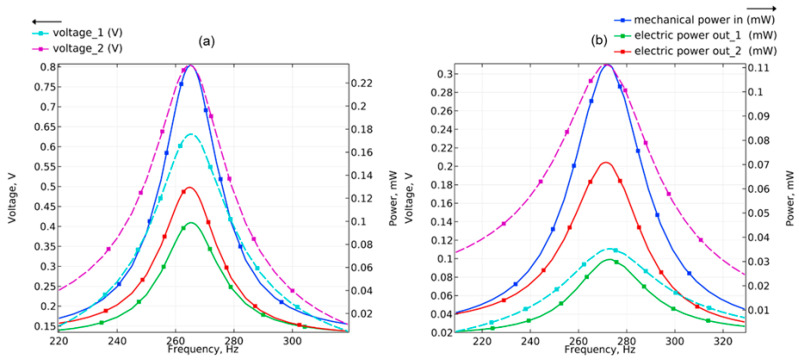
Frequency response of the cantilevers: voltage output, mechanical power, and electrical power as functions of frequency: (**a**) optimized design; (**b**) uniform thickness design. Arrow direction indicates the corresponding Y-axis for each legend.

Optimized and uniform thickness shape geometry piezoelectric beams were segment into two piezoelectric layers: first—from fixed end up to strain node and second—from strain node up to the free end. A further investigation was carried out through simulations of the piezoelectric cantilever beams. As in the previous analysis, the response of the optimized geometry (Figure 11a) was directly compared with that of the uniform beam (Figure 11b). The frequency response results presented in Figure 11 show voltage output (sky blue and magenta, dashed line), mechanical power (blue, solid line), and electrical power (green and red, solid line) as functions of frequency.

The optimized cantilever achieved peak voltage values of 0.63 V for the first piezoelectric layer and 0.81 V for the second piezoelectric layer, along with a maximum electrical power of 0.1 mW for the first piezoelectric layer and 0.13 mW for the second piezoelectric layer, whereas the uniform design produced significantly lower outputs—0.11 V and 0.31 V, respectively, with a maximum electrical power—0.03 mW and 0.07 mW, respectively. These results demonstrate a substantial improvement in electromechanical performance for the optimized configuration under second-mode excitation.

**Figure 12:**
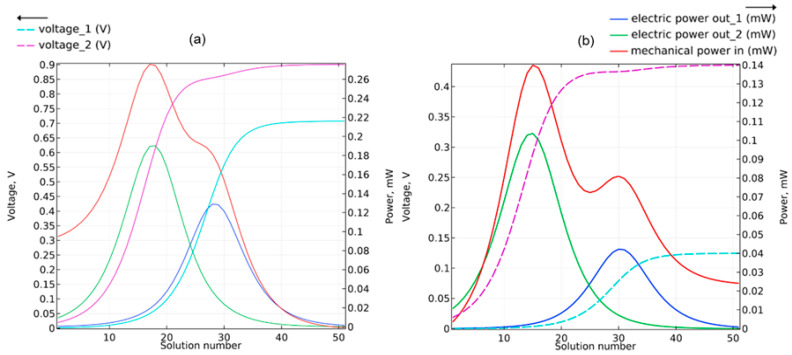
Load-resistance dependence of energy harvesting performance: voltage, mechanical power, and electrical power as functions of resistance: (**a**) optimized cantilever; (**b**) uniform thickness cantilever. Arrow direction indicates the corresponding Y-axis for each legend.

Figure 12 illustrates the load resistance dependence of energy harvesting performance for both designs, where voltage output (sky blue and magenta), mechanical power (red), and electrical power (green and blue) are plotted.

The horizontal axis, labeled as solution number, represents different solution iterations obtained using various resistor configurations, which are specified in Appendix A, Table A1, spanning a resistance range from 10 Ω to 10^7^ Ω. Each iteration corresponds to a distinct electrical load condition applied to the piezoelectric layers, allowing evaluation of power output sensitivity to load resistance.

The results indicate that the optimized cantilever achieves a maximum electrical power 0.13 mW at load resistances of R1 = 6.3 kΩ for the first piezoelectric patch and 0.19 mW at load resistances of R2 = 5.0 kΩ at the second piezoelectric patch. In contrast, the uniform (non-optimized) cantilever reaches its electrical power peak 0.04 mW performance at R1 = 7.9 kΩ and 0.1 mW at R2 = 2.0 kΩ for the corresponding patches, highlighting the influence of structural optimization on optimal electrical load matching and overall energy harvesting efficiency.

These results confirm that the optimized geometry significantly enhances strain generation in both active regions of the cantilever when operating at the second eigenfrequency. The redistribution of stiffness and inertia along the beam, combined with the relocation of the strain node, enables more efficient utilization of the cantilever length for energy conversion.

## 4. Experimental Validation and Discussion

Experimental studies were conducted to verify the numerical predictions and to evaluate the dynamic behavior of the cantilever optimized for operation at the second bending eigenfrequency. Two complementary experimental methods were used. First, a non-contact modal analysis based on multipath laser interferometry was performed to identify the resonance frequencies and vibration mode shapes of the fabricated cantilevers. Second, the electromechanical response of the structures was investigated using an impact-response excitation method, allowing the electrical output of the piezoelectric sensors to be compared with the strain distribution predicted by the finite element model.

Two cantilever configurations were produced for experimental comparison: a reference beam with uniform thickness and a beam with an optimized thickness distribution designed specifically for operation at the second vibration mode. Both structures were fabricated with identical planform dimensions in order to isolate the influence of thickness variation on the dynamic response.

The cantilevers were manufactured using a Formlabs Form 3L stereolithography printer (Formlabs, Somerville, MA, USA), which enabled accurate reproduction of the optimized geometry. The structural material used in both specimens was Engineering Resin Tough 2000(Formlabs, Somerville, MA, USA), a photopolymer characterized by stable mechanical properties and reliable post-curing performance. The material has a tensile modulus of approximately 2.2 GPa, a Poisson’s ratio of 0.41 [48], and a density of 1110 kg/m^3^.

Both cantilevers had identical in-plane dimensions of 100 mm in length and 10 mm in width. The reference cantilever had a uniform thickness of 1.8 mm along its entire length. The optimized cantilever exhibited a nonuniform thickness distribution ranging from 1.45 mm to 2.33 mm.

**Figure 13:**
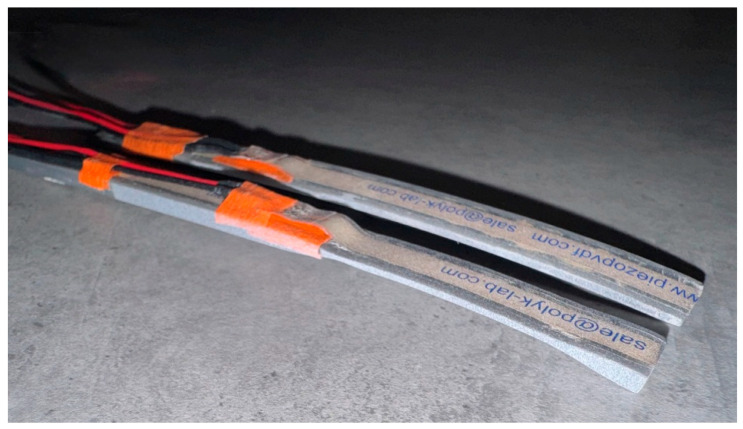
Illustration of the manufactured cantilevers—top cantilever is uniform thickness shape, and bottom cantilever is optimal shape.

The optimized structure can be interpreted as consisting of three functional regions. The first region extends from the clamped boundary to approximately 0.54 L and is characterized by an increased thickness close to 2.33 mm. Due to its proximity to the fixed support, this section experiences relatively low strain and primarily contributes to structural stiffness. The second region extends from approximately 0.54 L to 0.84 L and gradually narrows to the thinnest portion of the beam, reaching a thickness of approximately 1.45 mm. This segment forms the primary flexible region responsible for strain generation. The final region extends from 0.84 L to the free end and features a nonlinear increase in thickness up to approximately 2.1 mm, acting as an inertial section that increases the dynamic response of the system. Manufactured specimens are presented in Figure 13.

Finite element analysis indicated that the strain node associated with the second bending mode occurs at approximately 0.41 L along the beam length. To prevent cancellation of electrical charges produced by opposite strain polarities on either side of this node, the piezoelectric layer was divided into two independent sensing layers positioned on each side of the nodal point.

Each sensing element consisted of a 28 μm PVDF DT1-028K/L film (PolyK Technologies, State College, PA, USA) bonded to the upper surface of the cantilever. Electrical contacts were extended from each layer to enable independent measurement of the generated voltage signals.

Figure 13 shows the fabricated cantilever specimens used in the experiments.

### 4.1. Multipath Laser Interferometry for Modal Identification

**Figure 14:**
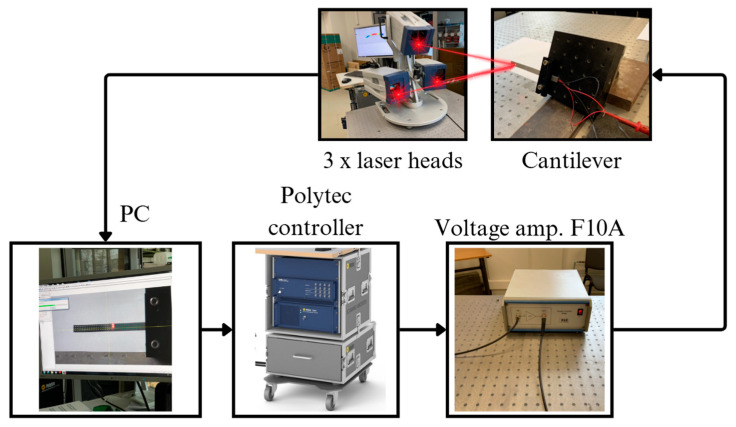
Schematic view of the experimental setup for amplitude-frequency characteristics.

The modal properties of the fabricated cantilevers were identified using a PSV QTec 3D scanning laser vibrometer system (Polytec, Irvine, CA, USA). The experimental setup consisted of a computer with control software, a vibrometer controller unit, a signal amplifier F10A (FLC Electronics, Partille, Sweden), the cantilever specimen fixed in a clamping fixture, and the laser vibrometer camera system. A schematic view of the experimental setup is shown in Figure 14.

During the measurements, the cantilevers were excited using short sinusoidal bursts applied through the piezoelectric layer, while the laser vibrometer recorded the resulting vibration velocities across the cantilever surface. The excitation frequency was swept over a range from 0 Hz to 1000 Hz in order to capture the resonance response of the structure.

A total of 114 measurement points were collected across an area of approximately 1000 mm^2^ on each cantilever. The measured vibration velocities were recorded along three orthogonal axes, where the out-of-plane motion in the Z-direction represented the dominant vibration component. The data were used to reconstruct the modal response and to identify the resonance frequencies corresponding to the vibration modes.

**Figure 15:**
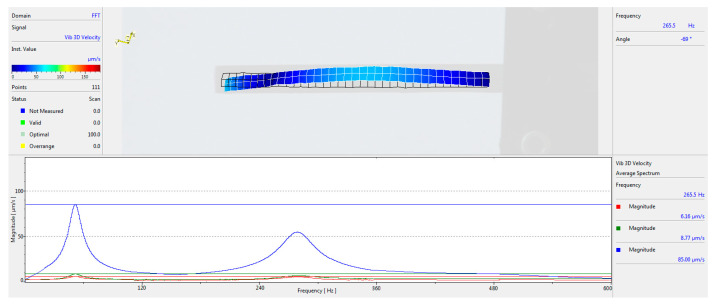
Amplitude–frequency characteristic of the cantilever of a uniform thickness.

**Figure 16:**
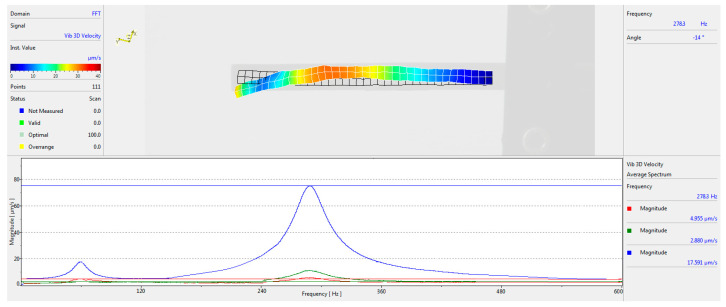
Amplitude–frequency characteristic of the cantilever of an optimally shaped thickness.

The interferometric measurements clearly revealed the modal shape associated with the second bending mode, including the presence of a nodal point along the beam where the vibration amplitude approaches zero. The experimentally observed modal pattern corresponded well with the predicted location of the strain node obtained from the numerical simulations. The results are shown in Figure 15 and Figure 16. A comparison reveals notable differences in the amplitude–frequency characteristics between the uniform-thickness and optimized cantilevers. In the uniform-thickness configuration, the amplitude at the second resonance remains lower than that of the first resonance, and the effective frequency response region is relatively narrow, approximately spanning from 180 Hz to 370 Hz. In contrast, the optimized cantilever exhibits significantly altered dynamic behavior, where the amplitude at the second resonance exceeds that of the first resonance. Moreover, the frequency response region of the second mode is considerably broader, extending approximately from 140 Hz to 480 Hz. These differences can be attributed to the modified geometry of the optimized cantilever, which redistributes stiffness and mass along the beam, leading to enhanced dynamic response and wider operational bandwidth.

The simulations and experimental measurements for the uniform thickness cantilever yielded second eigenfrequencies of 271.2 Hz in the numerical model and 265.5 Hz in the experimental measurements. The difference between these values was approximately 2.64%, indicating good agreement between the numerical predictions and the measured dynamic response. Similarly, for the optimized cantilever, the second eigenfrequencies were 271.4 Hz in the numerical model and 278.3 Hz in the experimental measurements, resulting in a difference of approximately 2.54%, which further confirms the accuracy of the developed model.

### 4.2. Electromechanical Response Under Constant Excitation

**Figure 17:**
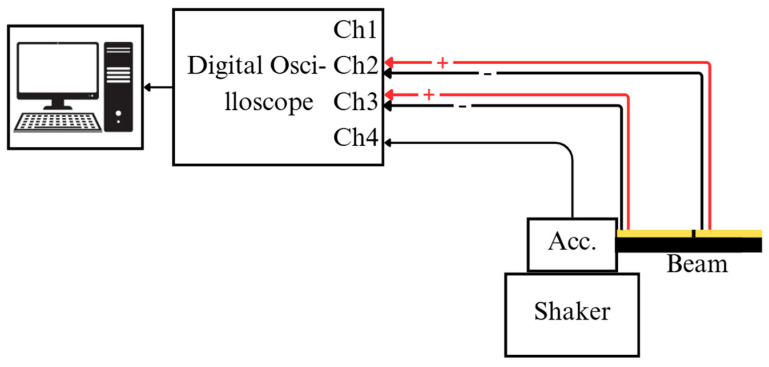
Schematic representation of the experimental setup used for amplitude–frequency characterization and voltage measurements.

The second experimental assessment was performed using a constant excitation approach, in which the cantilevers were driven by a vibrating platform. Under these conditions, the piezoelectric elements functioned in sensing mode, in contrast to their actuator role in the initial experimental configuration. Each specimen was rigidly mounted to the shaker via a clamping fixture, while the excitation signal was generated using a 33220A function generator (Keysight, Santa Rosa, CA, USA). To ensure sufficient signal quality, the excitation was amplified using a VPA2100MN voltage amplifier (HQ Power, Gavere, Belgium). The applied excitation level was monitored using a KS-93 single-axis accelerometer with a sensitivity of 5 mV/(m/s^2^). Measurement data from the accelerometer, piezoelectric elements, and laser control system were acquired using a 4224 USB oscilloscope (Pico Technology, St Neots, Cambridgeshire, UK) and subsequently processed in PicoScope software (Version 6.14). A schematic representation of the experimental arrangement is provided in Figure 17.

**Figure 18:**
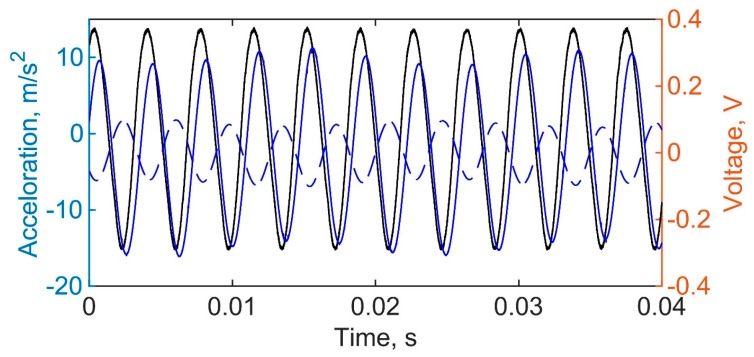
Experimental results of the uniform thickness shape cantilever: blue solid—output signal from the strain node to the tip of cantilever (right axis), blue dashed—output signal from the fixed boundary to the strain node of cantilever (right axis). Black—acceleration of the cantilever base (left axis).

**Figure 19:**
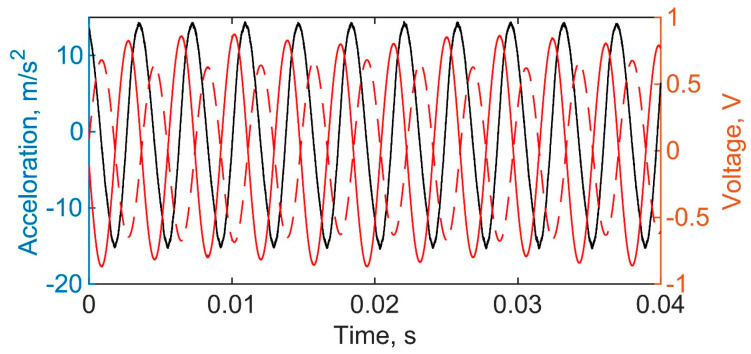
Experimental results of the optimal shape cantilever: red solid—output signal from the strain node to the tip of cantilever (right axis), red dashed –output signal from the fixed boundary to the strain node of cantilever (right axis). Black—acceleration of the cantilever base (left axis).

The results obtained from this second setup are presented in Figure 18 and Figure 19, where the excitation acceleration of the vibrating plate is depicted alongside the voltage responses of each cantilever segment under open-circuit conditions for the uniform thickness and optimized shape designs, respectively.

A direct comparison between the numerical and experimental voltage responses further confirms the consistency of the developed model. For the uniform-thickness cantilever, the numerical peak voltage amplitudes are approximately 0.11 V and 0.31 V for the first and second piezoelectric layers, respectively, while the corresponding experimental values are 0.10 V and 0.33 V, showing close agreement between the two, as presented in Figure 11b and Figure 18. The agreement between simulation and experiment is therefore very strong, with negligible deviation, particularly for the second layer. Similarly, for the optimized cantilever, the numerical results in Figure 11a indicate peak voltages of approximately 0.63 V and 0.81 V for the first and second piezoelectric layers. These values are in close agreement with the experimental results presented in Figure 19, where measured voltages reached 0.62 V and 0.78 V. The deviation between numerical and experimental values in this case remains minimal, confirming accurate prediction of electromechanical behavior.

Experimental measurements and statistical analysis of the voltage generated by the piezoelectric cantilevers were performed. During the experiment, both the optimized-thickness and uniform-thickness cantilevers were excited at their second resonance frequencies, and the voltages generated by the two piezoelectric patches were measured. The electrical load was 35 kΩ. Before each measurement, the cantilever wires were reconnected to the digital oscilloscope, and the cantilevers were reattached to the vibration platform to evaluate clamping condition variations. This procedure was repeated eight times for each cantilever. The measured voltage amplitudes are presented in Table 1.

Since such processes generally exhibit a Gaussian distribution, the application of the 2σ rule provides voltage prediction intervals with a confidence level of approximately 95%. The statistical parameters calculated from the experimental measurements are summarized in Table 2.

Furthermore, power outputs at different loads are evaluated and given in Table 3 for the optimal shape cantilever, as well as in Table 4 for the uniform shape one.

The experimental tests show that the optimized cantilever maximum electrical power is 0.11 mW at load resistances of R1 = 6.0 kΩ for the first piezoelectric patch and 0.16 mW at load resistances of R2 = 4.8 kΩ at the second piezoelectric patch. The uniform thickness cantilever electrical power peak is 0.03 mW at R1 = 7.7 kΩ and 0.11 mW at R2 = 2.3 kΩ for the corresponding patches.

This validates the reliability of the finite element model and confirms its capability to accurately capture the influence of thickness optimization on the electromechanical performance of cantilever-type piezoelectric energy harvesters.

## 5. Conclusions

This study presents a comprehensive modeling, optimization, and experimental validation framework for cantilever-type piezoelectric vibration energy harvesters operating at the second bending eigenfrequency. The developed approach is based on the Euler–Bernoulli beam theory and its finite element implementation, enabling accurate prediction of structural response and strain distribution. A gradient projection method in state space was employed to optimize the thickness distribution of the cantilever while constraining the second eigenfrequency, allowing enhancement of electromechanical performance without altering the global dimensions or dynamic characteristics of the structure. The formulation incorporates linear piezoelectric coupling and provides a systematic methodology for maximizing axial strain, which directly governs electrical energy generation.

The numerical results demonstrate that thickness redistribution significantly modifies the stiffness and inertia distribution along the beam, leading to a shift of the strain node and improved utilization of the cantilever length. The optimized design produces substantially higher strain levels across both active regions, resulting in an increase of more than two times in the integral strain compared to the uniform thickness configuration. Correspondingly, the electromechanical simulations indicate a significant improvement in voltage and power output, with the optimized cantilever reaching 0.23 mW of electrical power compared to 0.11 mW for the uniform design.

Experimental validation was carried out using stereolithography-fabricated specimens and two complementary measurement techniques, including laser interferometry for modal identification and controlled excitation tests for electromechanical response evaluation. The experimentally identified second eigenfrequencies were 265.5 Hz and 278.3 Hz for the uniform thickness and optimized cantilevers, respectively, compared to 271.2 Hz and 271.4 Hz predicted numerically, resulting in deviations of approximately 2.64% and 2.54%.

Furthermore, the measured voltage outputs demonstrated strong agreement with simulation results. The uniform cantilever produced 0.10 V and 0.33 V, while the optimized cantilever achieved significantly higher amplitudes of 0.62 V and 0.78 V.

Overall, the results demonstrate that thickness-based shape optimization is an effective strategy for enhancing the performance of cantilever-type piezoelectric energy harvesters, particularly for higher-mode operation. The proposed approach enables targeted improvement of strain distribution and energy conversion efficiency while maintaining compatibility with practical design constraints, making it suitable for real-world vibration energy harvesting applications.

## Figures and Tables

**Table 1:** Generated voltages.

Measurement Number	Voltage Generated by the Piezoelectric Cantilever, V			
	Optimized Thickness Sensor 1	Optimized Thickness Sensor 2	Uniform Thickness Sensor 1	Uniform Thickness Sensor 2
1	0.64	0.80	0.10	0.34
2	0.59	0.76	0.09	0.32
3	0.65	0.79	0.11	0.33
4	0.61	0.77	0.10	0.34
5	0.63	0.78	0.10	0.33
6	0.60	0.75	0.09	0.32
7	0.62	0.79	0.10	0.34
8	0.62	0.80	0.11	0.32

**Table 2:** Main statistical parameters of the unimorph cantilevers’ generated voltage.

Parameter	Mean, V	Variance	Standard Deviation
Optimized thickness—Sensor 1	0.62	0.50 × 10^−3^	0.22 × 10^−1^
Optimized thickness—Sensor 2	0.78	0.37 × 10^−3^	0.19 × 10^−1^
Uniform thickness—Sensor 1	0.10	0.50 × 10^−4^	0.71 × 10^−2^
Uniform thickness—Sensor 2	0.33	0.79 × 10^−4^	0.89 × 10^−2^

**Table 3:** Power output experimental measurements at different loads, Optimal shape cantilever.

Parameter	Mean						
R1, kΩ	1	2.3	4.8	6	7.7	10	20
P1, mW	0.041	0.89	0.109	0.11	0.098	0.087	0.057
R2, kΩ	1	2.3	4.8	6	7.7	10	20
P2, mW	0.069	0.138	0.16	0.159	0.148	0.136	0.076

**Table 4:** Power output experimental measurements at different loads, Uniform shape cantilever.

Parameter	Mean						
R1, kΩ	1	2.3	4.8	6	7.7	10	20
P1, mW	0.004	0.009	0.019	0.026	0.03	0.025	0.009
R2, kΩ	1	2.3	4.8	6	7.7	10	20
P2, mW	0.077	0.11	0.09	0.081	0.069	0.055	0.022

## Data Availability

The original contributions presented in the study are included in the article; further inquiries can be directed to the corresponding author.

## Appendix A

Load resistance solution number and parameters R1 and R2 values for initial shape and optimized cantilever.

**Table A1:** Load resistance value for each solution number.

Solution Number	R1 Load (Ω)	R2 Load (Ω)
1	10	100
2	12.589	125.89
3	15.849	158.49
4	19.953	199.53
5	25.119	251.19
6	31.623	316.23
7	39.811	398.11
8	50.119	501.19
9	63.096	630.96
10	79.433	794.33
11	100	1000
12	125.89	1258.9
13	158.49	1584.9
14	199.53	1995.3
15	251.19	2511.9
16	316.23	3162.3
17	398.11	3981.1
18	501.19	5011.9
19	630.96	6309.6
20	794.33	7943.3
21	1000	10,000
22	1258.9	12,589
23	1584.9	15,849
24	1995.3	19,953
25	2511.9	25,119
26	3162.3	31,623
27	3981.1	39,811
28	5011.9	50,119
29	6309.6	63,096
30	7943.3	79,433
31	10,000	10^5^
32	12,589	1.2589 × 10^5^
33	15,849	1.5849 × 10^5^
34	19,953	1.9953 × 10^5^
35	25,119	2.5119 × 10^5^
36	31,623	3.1623 × 10^5^
37	39,811	3.9811 × 10^5^
38	50,119	5.0119 × 10^5^
39	63,096	6.3096 × 10^5^
40	79,433	7.9433 × 10^5^
41	10^5^	10^6^
42	1.2589 × 10^5^	1.2589 × 10^6^
43	1.5849 × 10^5^	1.5849 × 10^6^
44	1.9953 × 10^5^	1.9953 × 10^6^
45	2.5119 × 10^5^	2.5119 × 10^6^
46	3.1623 × 10^5^	3.1623 × 10^6^
47	3.9811 × 10^5^	3.9811 × 10^6^
48	5.0119 × 10^5^	5.0119 × 10^6^
49	6.3096 × 10^5^	6.3096 × 10^6^
50	7.9433 × 10^5^	7.9433 × 10^6^
51	10^6^	10^7^
